# Marvin Minsky: The Visionary Behind the Confocal Microscope and the Father of Artificial Intelligence

**DOI:** 10.7759/cureus.68434

**Published:** 2024-09-02

**Authors:** Bhagyashri Patil-Takbhate, Rupesh Takbhate, Priyanka Khopkar-Kale, Srikanth Tripathy

**Affiliations:** 1 Central Research Facility, Dr. D. Y. Patil Medical College, Hospital and Research Centre, Dr. D. Y. Patil Vidyapeeth (Deemed to be University) Pimpri, Pune, IND

**Keywords:** artificial intelligence, historical vignette, neural network, the emotion machine, confocal laser scanning microscope

## Abstract

Marvin Lee Minsky, a pioneering figure in artificial intelligence (AI), was born on August 9, 1927, in the city of New York. His father, Henry, was an eye surgeon, while his mother, Fannie, was involved in Zionist activities. Minsky was instrumental in establishing the AI laboratory at the Massachusetts Institute of Technology (MIT) and authored numerous influential works on AI and philosophy. Among his many accolades was the prestigious Turing Award, which he received in 1969. Minsky was an exceptionally brilliant, creative, and charismatic individual, whose intellect and imagination were evident in his work. His ideas played a pivotal role in shaping the computer revolution that has profoundly transformed modern life in recent decades. In 1957, Minsky patented the confocal microscope, a significant invention that was a forerunner to today's confocal laser scanning microscopes. This innovation significantly improved image clarity and contrast by focusing light on a specific depth within a sample, unlike traditional microscopes, which allow light to penetrate deeper layers. The influence of his contributions continues to resonate in contemporary efforts to develop intelligent machines, one of the most thrilling and significant undertakings of our time.

## Introduction and background

Marvin Minsky (born on August 9, 1927, in New York and died on January 24, 2016, in Boston, Massachusetts) was an American mathematician [1] and computer scientist, one of the most famous practitioners of the science of artificial intelligence (AI). Minsky won the 1969 A.M. Turing Award, the highest honor in computer science, for his pioneering work in AI.

After serving in the US Navy during the Second World War, he pursued and obtained a mathematics degree from Harvard University in Cambridge, Massachusetts, in 1950. There he caught the attention of mathematician Andrew Gleason by successfully proving fixed-point theorems within the field of topology. While working on his doctoral studies concerning learning machines at Princeton University in New Jersey, he constructed a machine utilizing vacuum tubes and motors.

Upon completing his PhD, he received strong endorsements from renowned mathematicians John von Neumann, Norbert Wiener, and Claude Shannon, leading to his appointment as a junior fellow at Harvard. During his fellowship, his curiosity about the functioning of the human brain grew, but he encountered obstacles due to the limitations of conventional microscopy techniques, which failed to produce clear images of dense, light-scattering neural tissues. This challenge motivated him to invent the confocal scanning microscope, a device that employs lenses to concentrate light on progressively smaller volumes for improved imaging clarity.

Minsky was instrumental in establishing the AI laboratory at the Massachusetts Institute of Technology (MIT) and authored numerous influential works on AI and philosophy [1]. Among his many accolades was the prestigious Turing Award, which he received in 1969. Minsky was an exceptionally brilliant, creative, and charismatic individual, whose intellect and imagination were evident in his work. His ideas played a pivotal role in shaping the computer revolution that has profoundly transformed modern life in recent decades. In 1957, Minsky patented the confocal microscope, a significant invention that was a forerunner to today's confocal laser scanning microscopes [1]. This innovation significantly improved image clarity and contrast by focusing light on a specific depth within a sample, unlike traditional microscopes, which allow light to penetrate deeper layers. The influence of his contributions continues to resonate in contemporary efforts to develop intelligent machines, one of the most thrilling and significant undertakings of our time [2].

Long before the invention of the microprocessor and supercomputer, Professor Minsky, a highly respected computer science educator at MIT, laid the groundwork for the field of artificial intelligence by exploring how to instill common sense reasoning in computers [2].

"Marvin was among the few in computing whose visionary ideas transformed the computer from a mere advanced calculator into a tool that could potentially become one of the most powerful amplifiers of human endeavors in history," said Alan Kay, a computer scientist and close colleague of Professor Minsky.

From his undergraduate days at Harvard, Minsky was deeply intrigued by the mysteries of human intelligence and cognition. He believed there was no fundamental difference between human thinking and machine processes. Starting in the early 1950s, he developed computational theories to model human psychological processes and explored ways to equip machines with intelligence.

Marvin Minsky, a pioneering figure in artificial intelligence who merged a scientist's pursuit of knowledge with a philosopher's search for truth, passed away on Sunday, January 24, 2016, night, in Boston at the age of 88. His family reported that the cause was a cerebral hemorrhage.

## Review

Early life and education

Minsky earned a bachelor's degree in mathematics from Harvard University in 1950, followed by a doctorate in the subject of mathematics from the University of Princeton in 1954. His doctoral research focused on how neural networks could simulate brain functions, which laid the groundwork for much of his later work [1]. He attended the Bronx High School of Science, a high school having the greatest number of Nobel Prize winners of any secondary school in the world. Minsky began teaching at MIT in 1958 and remained there for the duration of his career. In 1959, he co-founded a laboratory with John McCarthy that would later evolve into the MIT Computer Science and Artificial Intelligence Laboratory. During his time at MIT, Minsky held several key academic positions, including Professor of Electrical Engineering and Computer Science and Professor of Media Arts and Sciences. He was also a very clever and mature pianist. Music and psychology remained his lifelong interest. Minsky also served in the United States Navy from 1944 during the Second World War. He married Dr. Gloria Rudisch, who is a physician, and had three children [2].

Key contributions

Confocal Microscope

Marvin Lee Minsky, a prominent cognitive scientist and one of the pioneers of artificial intelligence, did not directly contribute to the development or use of confocal microscopy. His work focused primarily on AI, cognitive psychology, and the philosophy of mind. Minsky's research often revolved around understanding human cognition and how to replicate aspects of it in machines. In 1957, Minsky patented the confocal microscope, a significant invention that was a forerunner to today's confocal laser scanning microscopes. This innovation significantly improved image clarity and contrast by focusing light on a specific depth within a sample, unlike traditional microscopes, which allow light to penetrate deeper layers. This technique is particularly valuable for producing three-dimensional images of microscopic structures. Minsky's interest in optics, which was influenced by his father, began at an early age and played a crucial role in this invention [3].

**Figure 1:**
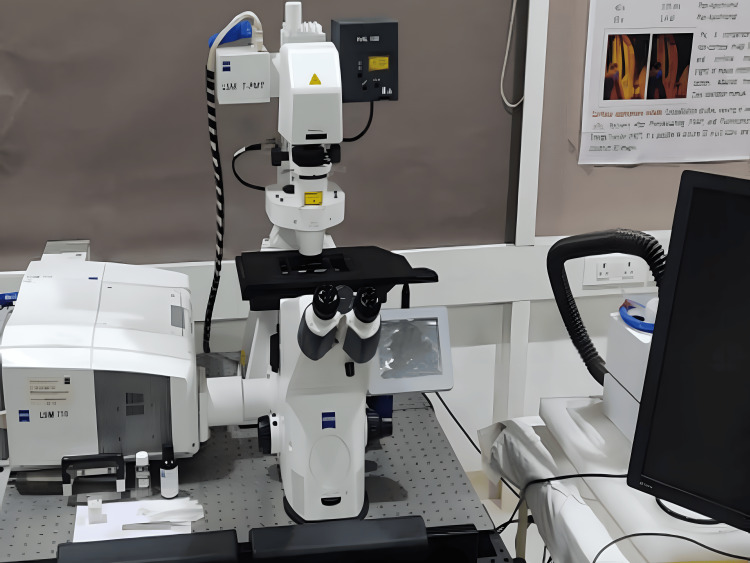
Zeiss 710 confocal laser scanning microscope Confocal microscope available at Dr. D. Y. Patil Medical College, Hospital and Research Centre, Pune

With advancements in precision instruments and semiconductor technology, the study of microstructure surface profiles has become increasingly important in scientific research. During the last few decades, confocal microscopy has emerged as a valuable tool in biological research. Despite the development of newer technologies such as light sheet and field synthesis microscopy, confocal microscopy remains essential in biological imaging due to its simplicity and broad accessibility [4]. For biologists with limited imaging experience, confocal microscopy-based methods offer an accessible solution for fundamental research, as more advanced technologies require significant expertise for effective use and analysis. By avoiding physical sectioning, tissues can be observed with the special ability of a confocal microscope that is optical or z-axis sectioning to further assimilate three-dimensional structure [3]. Nowadays, handy and affordable designs of confocal microscopy are currently under trial phase, which would be used in an in vivo manner for the early diagnosis of skin and cervical cancer [4]. The confocal microscopy model created by Marvin Minsky was very basic compared to today's advanced models. The model of the confocal laser scanning microscope available (Zeiss 710) at Dr. D. Y. Patil Medical College, Hospital and Research Centre, Pune is depicted in Figure 1.

Artificial Intelligence

Today's world is driven by artificial intelligence, and it has entered every field of human life. Marvin Minsky had a very clear and advanced vision for artificial intelligence. AI has now become a part of our daily lives [4]. Marvin Minsky defined artificial intelligence as "the science of making machines perform tasks that would require intelligence if done by humans." Although there were some early achievements, AI researchers struggled to encapsulate the complexities of the real world within the rigid syntax of the most advanced programming languages. In 1975, Minsky initiated the concept of "frames," a methodology to pinpoint the general knowledge that needs to be encoded in a computer before addressing specific instructions. Minsky's contributions to computer science were vast, including the creation of the first Logo "turtle" robot with Seymour Papert, which was used to teach children programming, and the development of SNARC, the first neural network learning machine, in 1951. In 1962, he worked on small universal Turing machines, publishing a renowned seven-state, four-symbol machine. His book "Perceptrons," co-authored by Papert, became a foundational text in the study of artificial neural networks, although it also sparked controversy and contributed to a slowdown in AI research during the 1970s. As AI has seen a resurgence in recent years, another aspect of Minsky's ideas might become increasingly relevant. Unlike the alarmist views on the potential dangers of AI, Minsky often adopted a more optimistic philosophical perspective on a future where machines could genuinely think. He believed that AI could eventually provide solutions to some of humanity's most significant challenges [5]. Dr. Minsky was a celebrity in the AI community. His name was also mentioned in famous books and movies such as "A Space Odyssey" in 2001.

Legacy

Marvin Minsky's contributions have left a legacy in both optical imaging and artificial intelligence. His invention of the confocal microscope has become a standard tool in research labs around the world, while his ideas about AI continue to influence ongoing research and development in the field. Overall, Minsky's career reflects a commitment to pushing the boundaries of scientific knowledge and technology, demonstrating how interdisciplinary innovation can lead to groundbreaking discoveries. Marvin Minsky, the physicist and inventor renowned for his development of the confocal microscope, passed away on January 24, 2016. In the final years of his life, he continued to be involved in his work and to share his ideas [6]. In his later years, Minsky remained active in his field and maintained a presence at MIT, where he continued to influence research and mentor younger scientists. Despite declining health, he was known to stay engaged with his professional interests and continued to contribute to discussions in his areas of expertise. Minsky received numerous accolades and recognition for his contributions to science and technology throughout his life. His death was widely mourned by the scientific community, with many colleagues and former students paying tribute to his significant contributions to both optical imaging and artificial intelligence. Minsky's legacy is enduring. The confocal microscope, which he developed, remains a crucial tool in biological and medical research. His pioneering work has had a lasting impact on how scientists visualize and analyze complex structures at the microscopic level.

Other Achievements of Marvin Minsky

Minsky introduced innovative ideas on knowledge representation in computers and speculated on the possibility of communicating with extraterrestrial life that might think like humans. In the early 1970s, he and Papert developed the "Society of Mind" theory, which proposed that intelligence arises from the interactions of many simple, non-intelligent parts. This theory was influenced by Minsky's work with a machine that used a robotic arm, video camera, and computer for building structures with children's blocks. He later expanded on these ideas in his 1986 book "The Society of Mind." Both Minsky and Papert were part of the broader intellectual environment at MIT, where interdisciplinary collaboration was encouraged [7]. While Papert focused on educational technology and programming, Minsky's work in AI and cognitive science complemented and influenced the development of related technologies and educational approaches [5]. Papert's primary contribution to the Logo turtle was through his work on the Logo language and his educational philosophy. Minsky's contributions were more focused on AI and cognitive theories, and while his work did not directly involve the Logo turtle, his influence on the broader field of computer science and education was significant. In summary, Seymour Papert was directly responsible for the creation of the Logo turtle and the development of the Logo programming language, while Marvin Minsky's contributions were more focused on AI and cognitive science. Both played important roles in shaping the field of educational technology, but their specific contributions were distinct. In 2006, Minsky released another influential book titled "The Emotion Machine," which delves into concepts such as consciousness, emotions, different levels of thinking, and common sense. A central theme throughout the book is multiplicity [5]. Minsky argued that our versatile intelligence stems from various modes of thinking, including search, analogy, divide and conquer, contradiction, elevation, simulation, reformulation, logical reasoning, and impersonation [8]. Minsky even conceptualized a "gravity machine," designed to ring a bell if the gravitational constant ever changed, although this was more of a theoretical exercise than a practical invention. During the 1980s, Marvin Minsky and IJ Good demonstrated how artificial neural networks could be created and replicated automatically, suggesting that artificial brains could evolve in a manner like human brains.

Marvin Minsky: Honors and awards

Marvin Minsky received many awards such as the Turing Award for Computing Machinery (1970), Lecturer in Smithsonian Institution (1978) and also Messenger Lecturer at Cornell University (1979), Killian Award, MIT (1989), Japan Prize Laureate (1990), Research Excellence Award, IJCAI (1991), Joseph Priestley Award (1995), Rank Prize, Royal Society of Medicine (1995), IEEE Computer Society Computer Pioneer Award (1995), R. W. Wood Prize, Optical Society of America (2001), Benjamin Franklin Medal, Franklin Institute (2001), In Praise of Reason Award, World Skeptics Congress (2002), Fellow, Computer History Museum (2006), Inducted into the IEEE Intelligent System's Hall of Fame for "significant contributions to the field of AI and intelligent systems" (2011), and Dan David Prize (2014) [6]. He served as a former President of the Artificial Intelligence Association of America and was a fellow of various prestigious academies [7]. Yale University awarded Minsky an honorary Doctor of Science degree, reflecting his influential contributions to the fields of science and technology. The University of Edinburgh conferred an honorary degree on Minsky, recognizing his impact on the field of artificial intelligence and his contributions to scientific research.

Marvin Minsky passed away in January 2016 at the age of 88 from a cerebral hemorrhage, but his groundbreaking work continues to influence and inspire the field of artificial intelligence. He will be always remembered for his central role in constructing, framing, recommending, and improving the area of artificial intelligence [8]. As intelligent devices become more common in our homes and autonomous vehicles enter our roads, it seems that AI will increasingly shape our lives, largely due to the insights and ingenuity of Marvin Minsky [9,10].

His laboratory was an egalitarian haven, where he paid no attention to appearance, gender, age, or status, only ideas and ability mattered to him. Minsky and his wife, Gloria, frequently invited students into their home, where the presence of several pianos served as a testament to Minsky's musical talent, as he was a prodigy capable of improvising fugues [11,12].

## Conclusions

Marvin Minsky, the cognitive scientist, is renowned for his work in AI, robotics, and cognitive theory, and his contributions are to the development of confocal microscopy. Marvin Minsky, the physicist and inventor who developed the confocal microscope, had a fascinating life and career with several key insights and contributions. Although he passed away, he will always be remembered for his crucial role in constructing, framing, recommending, and improving the area of artificial intelligence. As intelligent devices become more common in our homes and autonomous vehicles enter our roads, AI will increasingly ease our lives, largely due to the insights and ingenuity of Marvin Minsky.

We salute to such a prominent figure in medical science who made our lives easy with artificial intelligence.
